# AVD-YOLOv5: a new lightweight network architecture for high-speed aortic valve detection from a new and large echocardiography dataset

**DOI:** 10.1007/s11517-024-03090-3

**Published:** 2024-04-18

**Authors:** Mervenur Çakır, Murat Ekinci, Elif Baykal Kablan, Mürsel Şahin

**Affiliations:** 1 Software Engineering, Karadeniz Technical University, Trabzon, 61080 Turkey; 2 Computer Engineering, Karadeniz Technical University, Trabzon, 61080 Turkey; 3 Department of Cardiology, Faculty of Medicine, Karadeniz Technical University, Trabzon, 61080 Turkey

**Keywords:** Cardiac imaging, Echocardiography, Aortic valve, Object detection, Depth-wise separable convolution, YOLOv5

## Abstract

**Abstract:**

Heart disease detection is currently gaining widespread attention as a means to enhance the accuracy of cardiologists’ diagnoses from cardiac images and reduce diagnosis time. Although high-resolution computed tomography (CT) images are typically favored for heart disease detection, the drawbacks of cost and radiation exposure to patients necessitate alternative approaches. In this context, utilizing ultrasound images becomes pivotal to mitigate radiation risks and maintain cost-effectiveness. In this paper, we propose a novel lightweight model, AVD-YOLOv5, designed for automated aortic valve detection on echocardiography images. This model incorporates several enhancements to the YOLOv5 architecture. Notably, the depth-wise separable convolution significantly contributes to the model’s lightweight design by reducing the number of parameters while maintaining precision. We have also created a new and larger dataset comprising 260 echocardiography images specifically for aortic valve detection. Experimental results indicate that the precision value of the modified ADV-YOLOv5 model stands at 94.3%, with a recall value of 86.8%. The model also demonstrates a notable 67% reduction in inference time compared to the original YOLOv5 model. Although there is a marginal reduction in precision by 0.94%, the model’s efficiency is significantly increased. The proposed system can be used by cardiologists for more efficient and reliable diagnosis.

**Graphical abstract:**

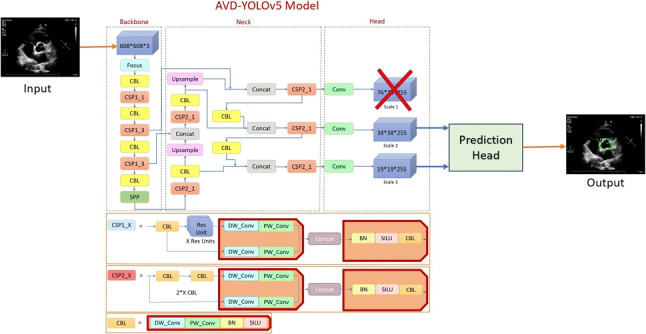

## Introduction

**Fig. 1:**
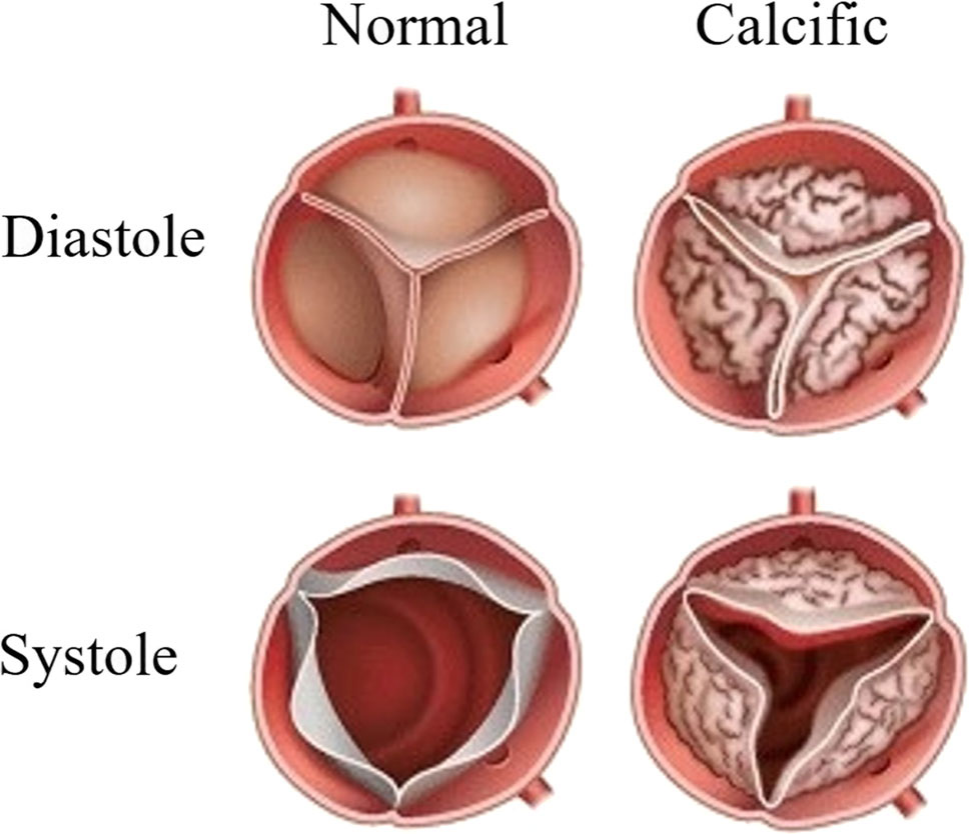
Illustration of normal and calcific aortic valve during systole and diastole phases of the heart muscle [7]

The aortic valve is located between the left ventricle and the aorta and prevents the blood normally ejected from the left ventricle into the aorta from returning to the left ventricle [1]. It normally consists of three leaflets. These three leaflets open when the heart contracts and close when it relaxes. This coordination allows blood to be distributed throughout the body without escaping back to the heart [2, 3]. Many heart valve diseases affect the normal function of the aortic valve [4]. The most common of these diseases is aortic stenosis, in which the aortic valve, which normally opens fully and allows blood to pass forward, narrows for various reasons and prevents the blood from passing forward [3]. The common cause of aortic stenosis in developed countries with a higher average age is calcification of the valves with age [5, 6]. Figure 1 shows the appearance of the normal and calcific aortic valve during the systole and diastole phases of the heart muscle. In the normal aortic valve view, there is no calcification on the three leaflets; the leaflets are thin and well-circumscribed. The leaflets in the calcific valve view are thick and irregularly circumscribed with calcification formation.

The expert cardiologists evaluate the calcification formation on the echocardiography image only on the three leaflets that make up the aortic valve. They therefore focus on the aortic region of interest (ROI) and then generate an approximate score for calcification volume and score. This manual evaluation performed by expert cardiologists has several disadvantages:

- The assessment of the aortic valve and its three leaflets is limited by speckle noise and low contrast on echocardiography images [8].
- The interpretation of images can be subjective and this leads to inconsistencies in calcium score predictions between cardiologists, affecting the accuracy and reliability of assessments [9].
- Even an expert cardiologist can make mistakes and lead to misdiagnosis due to factors such as fatigue and distraction during manual assessment.
- Since manual assessment is a time-consuming process, it may delay treatment plans.
- Expert cardiologists may not be available in all health facilities, especially in remote and underserved areas [10]. This may result in a delay in calcium score estimation for patients.

To address these disadvantages, automatic aortic valve detection methods are being developed to provide more objective, consistent, and efficient calcium score predictions in aortic valve assessments. It is also worth noting that most of these methods are based on deep learning, which has been boosted by the increasing popularity and power of deep learning techniques in recent years. Deep learning-based methods can automatically learn relevant features from raw data and achieve high levels of accuracy in tasks such as image classification, object detection, and segmentation. This accuracy is crucial in medical diagnosis, where even a small error can have significant consequences. The existing aortic valve detection methods [11–14] have shown promising results using various convolutional neural network (CNN)-based architectures. The literature review revealed that only a limited number of methods have been proposed for this specific task, which are presented below.

Nizar et al. [11] trained single shot multibox detector (SSD) and faster regional-based convolutional neural network (Faster R-CNN) object detector architectures with different feature extractors on a dataset including 23 echocardiography images from different patients. They evaluated the performance of the models on five echocardiography videos. With the Faster R-CNN object detector and Inception v2 feature extractor, they achieved the highest accuracy of 94.9%. In the second place, they achieved 86.5% accuracy with the SSD object detector and Inception v2 feature extractor. In terms of prediction speed, they emphasized that SSD architectures are faster. The results demonstrate the potential of using CNN-based architectures in the field of echocardiography and lay the foundation for further research in this area. Nizar et al. [12] then trained their proposed CNN-based object detector on a more advanced dataset from 33 different patients and performed a performance analysis on ten echocardiography videos. They obtained the highest accuracy of 98.6% with the Faster R-CNN object detector and Inception v2 feature extractor. They also reported the lowest graphics processing unit (GPU) utilization with SSD object detector and Mobilenet v2 feature extractor. Lai et al. [13] trained the AlexNet model on a dataset of 120 patient samples. The model achieved 95% accuracy in aortic valve detection. Hatfaludi et al. [14] detected the aortic valve in parasternal long-axis echocardiography images with a Faster R-CNN object detector with 93.3% accuracy and nearly 100% precision.

The studies discussed above have shown that CNN-based object detection models provide promising results in automatic aortic valve detection. These findings provide a solid foundation for the development of more sophisticated and accurate models in the field of echocardiography, which could have important implications for the diagnosis and management of cardiovascular diseases such as aortic stenosis. However, it is important to note that the aforementioned studies have certain limitations regarding the datasets they utilize. Although the proposed models have shown promising accuracy, generalizing these results to a larger and more diverse dataset may raise concerns. Therefore, further research and evaluation on larger and more diverse datasets are required to effectively validate and generalize the results.

CNN-based object detection models are divided into two groups: region-based models and single-stage models [15]. Region-based object detection models (R-CNN, Fast R-CNN, and Faster R-CNN) are implemented in two steps: (i) generation of region proposals and (ii) classification of these regions. In single-stage object detection models (SSD and You Only Look Once (YOLO) object detection models (YOLO [16], YOLO900 [17], YOLOv3 [18], YOLOv4 [19] and YOLOv5 [20])), both region recommendation and classification are performed in a single step. The main feature of YOLO architectures is that this single-stage detection approach is designed to detect objects in real time and with high accuracy. Unlike two-stage detection models, which first propose regions of interest and then classify these regions, YOLO processes the entire image in a single pass, making YOLO architectures faster and more efficient.

YOLO [16], the first proposed YOLO model, used a single CNN to detect objects belonging to different classes in the image and provided very fast detection compared to other two-stage object detection models. On the other hand, it was not as successful as some two-stage models at the time. YOLOv2 [17] proposed anchor boxes to improve the detection accuracy of the YOLO model and the UpSample layer that improves the resolution of the output feature map. YOLOv3 [18] introduced the Darknet-53 architecture, a derivative of the residual network (ResNet) architecture specifically designed for object detection, as the main improvement to previous models to increase the accuracy and speed of the algorithm. Also in YOLOv3, anchor boxes have been improved so that the size and shape of detected objects can be better matched using different scales and aspect ratios. The use of Feature Pyramid Networks (FPN) and the Gradient Harmonized Mechanism (GHM) loss function allows detection over a wide range of object sizes and aspect ratios, while accuracy and stability are significantly improved. YOLOv4 [19] introduced many improvements to the YOLOv3 model. These enhancements include a new backbone network, improvements to the training process, and increased model capacity. YOLOv4 also introduced the Cross Mini-Batch Normalization method designed to improve the stability of the training process. YOLOv5 [20] is a version released as an open-source project by Ultralytics in 2020, building on the success of previous versions. YOLOv5 utilized the EfficientDet architecture using the EfficientNet network as the backbone. It has also been enhanced to achieve improved object detection performance with many new features and improvements. With its flexible Python-based structure, YOLOv5 has become the world’s leading repo for object detection in 2020. In our previous study, we applied four sub-versions of the YOLOv5 model on a small aortic valve detection dataset consisting of 202 parasternal short-axis echocardiography images from 100 different patients and obtained 99.9% precision and 97.5% recall with the YOLOv5-x model [21].

Previous studies have mostly focused on improving the accuracy of aortic valve detection. On the other hand, rapid and accurate diagnosis can prevent disease progression and improve treatment success by detecting the disease at an early stage and starting the treatment process early. Hence, high speed certainly cannot be ignored as it has great practical value, especially in maritime emergency rescue and salvage operations in war emergency scenarios. Therefore, to address this problem, a novel high-speed aortic valve detection approach (AVD-YOLOv5) is proposed in this paper, mainly using a depth-wise separable convolution neural network (DS-Conv). A DS-Conv consists of a depth-wise convolution (DW_Conv) and a point-wise convolution (PW_Conv), which replaces the standard convolution neural network (Conv), and the number of network parameters is greatly reduced. To confirm the accuracy and feasibility of the proposed method, a new and larger dataset is proposed as there is no publicly available aortic valve detection dataset. Experimental results have shown that our method provides high-speed and successful aortic valve detection compared to state-of-the-art methods.

The main contributions of our work are as follows:

- To efficiently validate and generalize the results, a new and larger dataset of 260 echocardiography images for aortic valve detection was created.
- A new lightweight network architecture, AVD-YOLOv5, was developed for high-speed aortic valve detection mainly based on a depth-wise separable convolution neural network (DS-Conv) architecture.
- Extensive ablation studies and experiments on the aortic valve detection dataset indicate the effectiveness of the proposed method. It yielded faster results than other state-of-the-art object detectors without any degradation in detection accuracy.
- This is the first study that applies YOLO approaches for aortic valve detection and proposes an improved version of YOLOv5 architecture for this purpose.

The remainder of this paper is organized as follows. Section 2 introduces the standard YOLOv5 architecture and the proposed AVD-YOLOv5 architecture. Section 3 presents the experimental setup and its results. It also presents an extensive ablation study that highlights the contributions of various parts of the proposed architecture. Finally, evaluation and future work are given in Section 4.

**Fig. 2:**
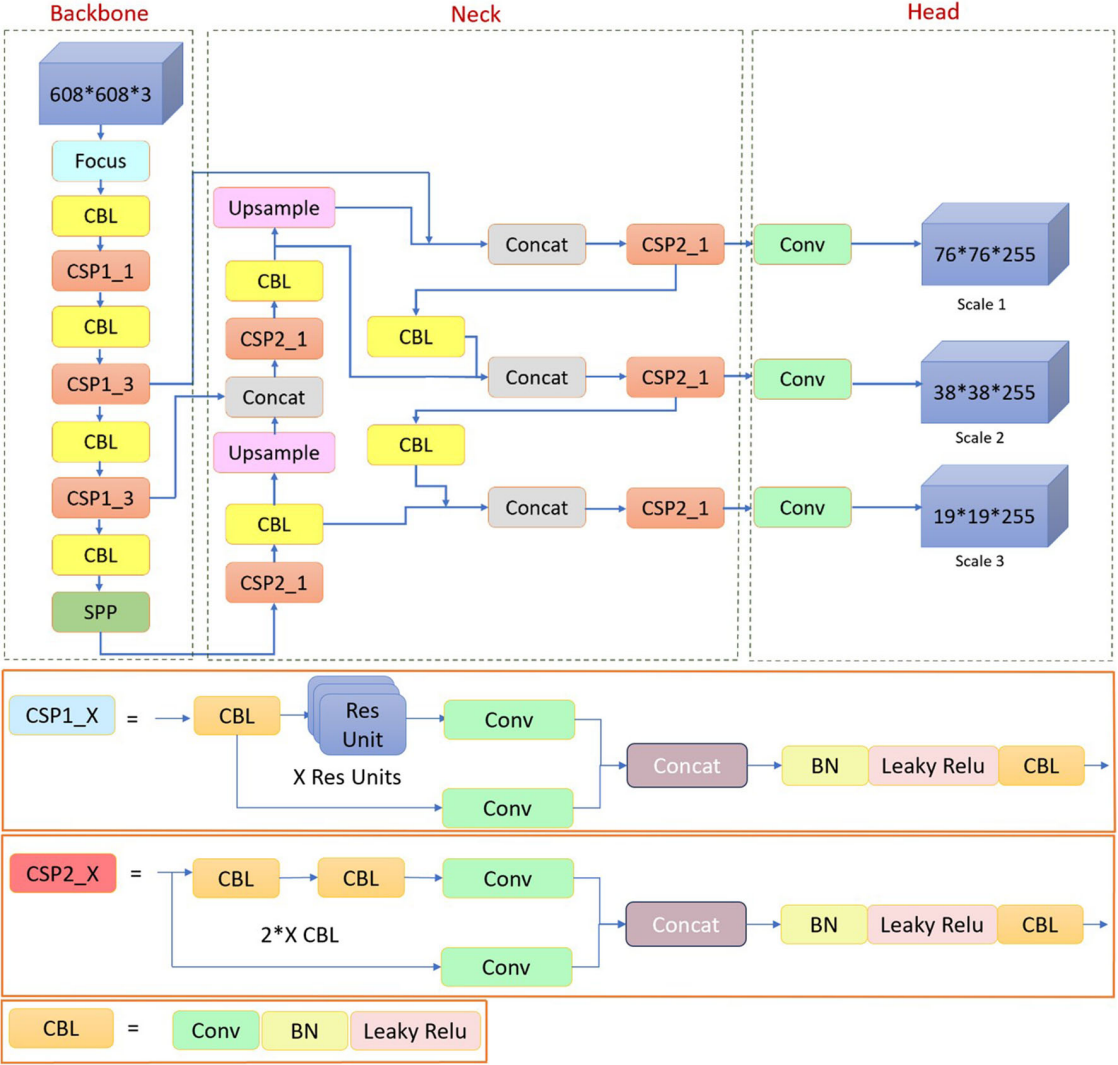
The general architecture overview of the YOLOv5 model. The model comprises three main components: backbone, neck, and output

## Methodology

### Overview of YOLOv5 model

The YOLOv5 [20] architecture is designed based on a network structure consisting of three main parts: backbone, neck, and head, as shown in Fig. 2. The backbone is used to extract key features from a given input image. A feature map is created using the extracted features. It is designed based on the Cross Stage Partial Network (CSPNet) [22] architecture, which aims to extract high-level features while providing high accuracy and reducing the processing time of the models. The neck collects feature maps from different stages of the model backbone to build feature pyramids. It uses the Path Aggregation Network (PANet) architecture to obtain feature pyramids [23]. The contribution of feature pyramids is to help the model detect objects of the same type at different sizes and scales. The head, the final object detection part of YOLOv5, is designed on the same architecture as YOLOv3 and YOLOv4. It applies anchor boxes to the final feature maps and generates the final output vectors with objectness scores, class belonging probabilities, and coordinates of the bounding boxes surrounding the detected objects. As shown in Fig. 2, the YOLOv5 backbone, neck, and head parts consist of many key modules. We can summarize these modules as follows.

**Table 1:** Network depth comparison of YOLOv5 sub-versions consisting of different CSP module structures

Module type	YOLOv5 sub-versions			
	YOLOv5-s	YOLOv5-m	YOLOv5-l	YOLOv5-x
Head (CSP1_X)	CSP1_1	CSP1_2	CSP1_3	CSP1_4
	CSP1_3	CSP1_6	CSP1_9	CSP1_12
	CSP1_3	CSP1_6	CSP1_9	CSP1_12
Backbone (CSP2_X)	CSP2_1	CSP2_2	CSP2_3	CSP2_4
	CSP2_1	CSP2_2	CSP2_3	CSP2_4
	CSP2_1	CSP2_2	CSP2_3	CSP2_4

**Focus module:** It divides the input image into four parallel slices with the same aspect ratio and then merges these slices on a depth basis. It passes the output to the CBL module to generate feature maps.

**CBL (convolutional block layer) module:** It is a basic-level feature extraction module consisting of convolution (Conv), batch normalization (BN), and leaky rectified linear unit (LeakyReLU) activation function, respectively.

**CSP (cross stage partial) module:** It is a CSPNet-based module that aims to improve the learning capacity of the model by extracting higher-level features. YOLOv5 includes two types of CSP modules, CSP1_X and CSP2_X. The CSP1_X module is used in the backbone part while the CSP2_X module is used in the neck part. CSP1_X contains one CBL module and X RES (residual) blocks. CSP2_X contains X$+$1 CBL module. More RES blocks and CBL modules increase the depth of the network architecture. Table 1 shows the CSP module structures used in the four sub-versions of the YOLOv5 model.

**SPP (spatial pyramid pooling) module:** It is used in the backbone to mix and pool spatial features. It combines input features with initial features after downsampling them through three parallel maximum pooling layers.

### Proposed AVD-YOLOv5 model

**Fig. 3:**
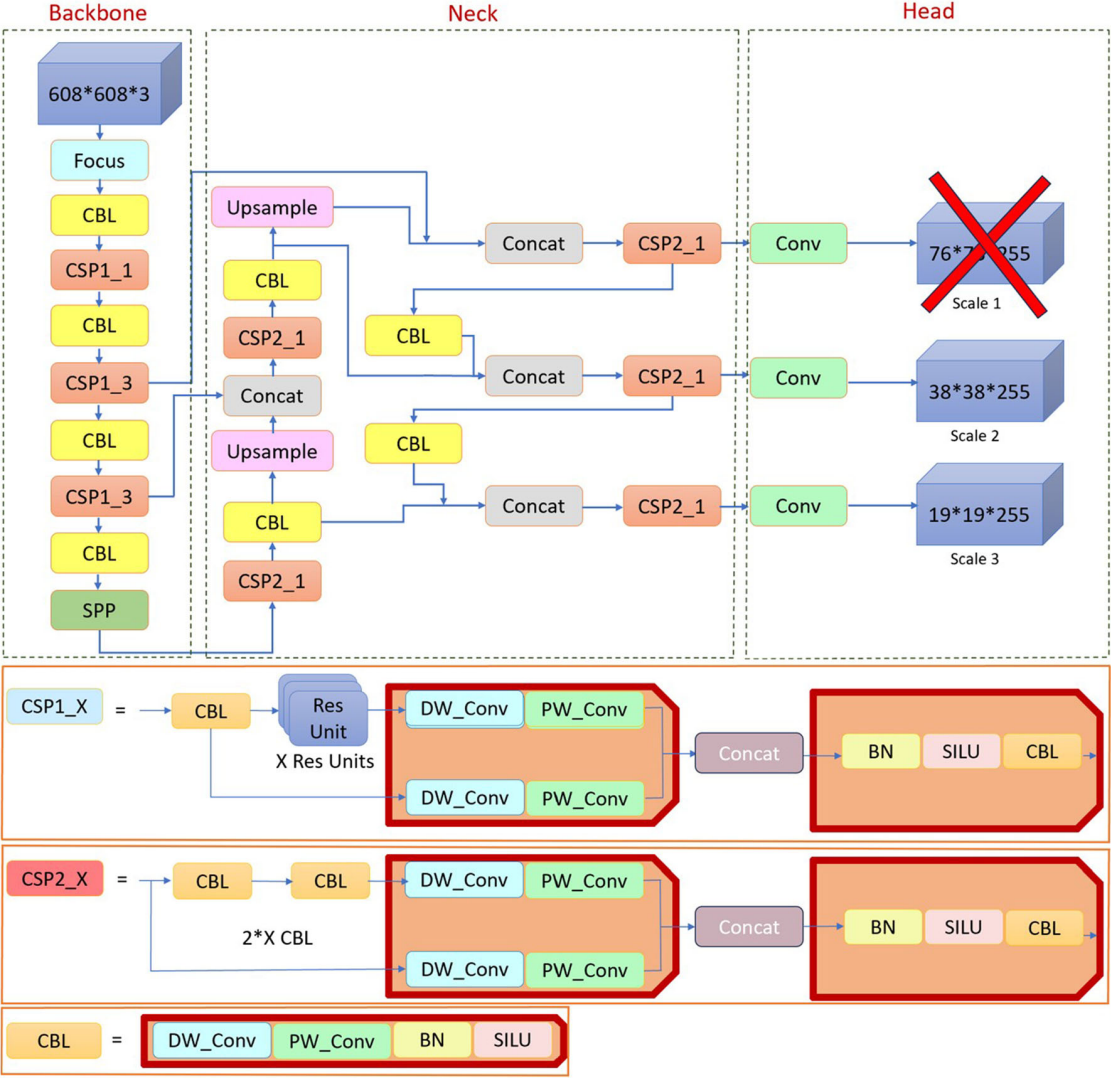
The general architecture of the proposed AVD-YOLOv5 model. It uses depth-wise separable convolution (DS-Conv) in CSP and consequently, CBL blocks which consist of a depth-wise convolution (DW_Conv), and a point-wise convolution (PW_Conv)

We propose the AVD-YOLOv5 model for high-speed aortic valve detection from echocardiography images. As shown in Fig. 3, we have made several improvements to the original YOLOv5 architecture: (1) small-scale detection is excluded from the configuration file since the aortic valve region of interest (ROI) does not contain excessively small details relative to the size of the whole input image; (2) depth-wise separable convolution (DS-Conv) is used in CSP blocks (CSP1_X and CSP2_X), which is different from the standard convolution process; (3) instead of using the available anchor boxes, a *K*-means algorithm is used to determine the size of the anchor boxes that best fit the searched object (aortic valve).

#### Eliminating small-scale detection component (Scale 1) from the head

The terms “Scale 1,” “Scale 2,” and “Scale 3” given in the head section in Fig. 3 refer to the different levels of feature maps generated by the network for object detection at different resolutions. Scale 1 refers to the first scale of feature maps, which typically has the highest resolution. Scale 2 refers to the second scale of feature maps, which has a lower resolution compared to Scale 1. Scale 3 refers to the third scale of feature maps, which has an even lower resolution compared to Scale 2. Hence, Scale 1 will have a resolution of 76$\times$76, Scale 2 38$\times$38, and Scale 3 19$\times$19. The feature maps at these different scales are used to detect objects of different sizes in the input image. Relatively small-size objects will be better detected at Scale 1, while medium-size and large-size objects will be better detected at Scale 2 and Scale 3, respectively. The use of multiple scales helps YOLOv5 to efficiently handle objects of various sizes—small, medium, and large—in object detection tasks. However, in the case of the aortic valve region, there appear to be no excessively small details or objects to be detected. The aortic region mainly contains structures and features of reasonable size compared to the whole input image. Therefore, the small-scale detection component (Scale 1) is excluded in the configuration file of YOLOv5 for this specific application. In this way, the YOLOv5 model is optimized to focus on detecting larger and more important features in the aortic region, resulting in better overall performance and computational efficiency.

#### Replacing standard convolutions with depth-wise separable convolutions in CSP blocks

**Fig. 4:**
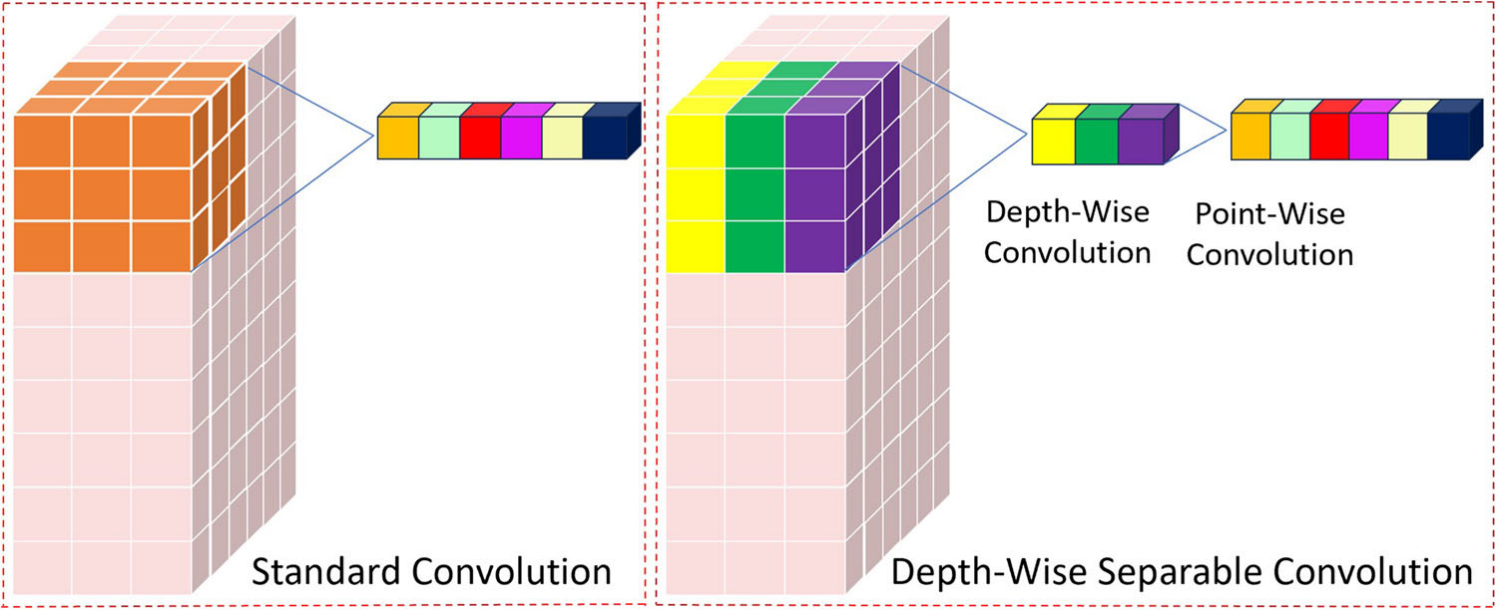
The standard convolution with 3$\times$3 kernel and three input channels shows the projection from 3$\times$3$\times$3 (dark blue) input values to six colored outputs with six output channels. In the depth decomposable convolution with 3$\times$3 kernel and three input channels, a depth convolution projects 3$\times$3 pixels of each input channel to a corresponding output pixel (matching colors). The 1$\times$1 point-wise convolution then uses these 3 output pixels to determine 6 final output pixels

To reduce computational cost and model size while maintaining reasonable performance, depth-wise separable convolution, proposed in MobileNet [24], is often used instead of standard convolution. This is because depth-wise convolution is highly efficient in terms of the number of parameters and computations required. A comparison between a standard convolution and a depth-wise separable convolution is illustrated in Fig. 4.

Depth-wise separable convolution (DS-Conv) proposes to separate spatial interactions (image height and width) from channel interactions (e.g., colors). The standard convolution does this by multiplying values over both a few spatial pixels and all channels together, whereas the DS-Conv first uses a spatial-only and channel-independent depth-wise convolution (DW_Conv). In practice, this means that we have an independent convolution for each channel. We then apply a point-wise convolution (PW_Conv) that models the channel interactions. The PW_Conv uses a kernel of size 1$\times$1, so it does not model any spatial interactions.

**Table 2:** Default anchor boxes and generated anchor boxes for aortic valve detection using K-means clustering algorithm

Object scale	Default anchor boxes	Generated anchor boxes
19 (P3/8 )	[10,13, 16,30, 33,23]	[91,88, 98,85, 99,100]
38 (P4/16 )	[30,61, 62,45, 59,119]	[108,96, 109,111, 115,106]
76 (P5/32)	[116,90, 156,198, 373,326]	[128,108, 120,116, 134,124]

Assume that the size of the image is $W\times H$, the size of the convolution filter is $K\times K\times Cin$, and the number of convolution filters is *Nconv*. Then, the computational cost of standard convolution (Conv) is expressed as in Eq. 1.

1 $$\begin{aligned} Cost_{Conv} = H *W *K *K *Cin *Nconv \end{aligned}$$

If we use depth-wise separable convolution, the computational cost is the sum of the depth-wise convolution (DW_Conv) and the point-wise convolution (PW_Conv) as given in Eq. (2–4).

2 $$\begin{aligned} Cost_{DW_Conv}= & {} H *W *Cin *K *K \end{aligned}$$

3 $$\begin{aligned} Cost_{PW_Conv}= & {} H *W *Cin *Nconv \end{aligned}$$

4 $$\begin{aligned} Cost_{DS-Conv}= & {} Cost_{DWConv}+Cost_{PWConv} \end{aligned}$$

5 $$\begin{aligned} Cost_{DS-Conv}= & {} H *W *Cin *K *K + H *W \nonumber \\{} & {} *Cin *Nconv \end{aligned}$$

Then, the computational ratio of Conv and DS-Conv is given in Eq. (6), showing that the use of DS-Conv effectively reduces the computational cost and increases the detection speed.

6 $$\begin{aligned} \frac{Cost_{DS-Conv}}{Cost_{Conv}}=\frac{1}{Nconv}+\frac{1}{K^2} \end{aligned}$$

#### Generation of best anchor boxes for aortic valve detection task using *K*-means

Anchor boxes are the prediction boxes in the head component of the YOLOv5 architecture and are assigned to the possible coordinates of the searched object. A total of nine anchor boxes are calculated in each image, three for each scale. The box with the highest probability value is transferred to the output. Initially, the scales of the boxes in each network were determined by default. However, the content and structure of the images in different datasets are not fully compatible with random box scales. In this study, the *K*-means clustering algorithm was applied to the aortic valve dataset, and the sizes of the anchor boxes that best fit the searched object were determined.

The *K*-means algorithm is used to divide the object sizes into clusters and to find the cluster centers. When applying the algorithm, the initial centers are chosen randomly. The distance metric is usually the Euclidean distance. In the assignment step, each object is assigned to the nearest center. In this step, the dimensions of each object and the distance between the centers are calculated. In the center update step, new centers are calculated for each cluster based on the sizes of the objects within the cluster. Then, the assignment step is repeated, and the assignment of objects to centers is updated. The algorithm is finished at a point where the change in centers becomes very small, i.e., convergence. The resulting cluster centers (anchor boxes) represent the sizes of the objects in the training data set. This provides anchor boxes with appropriate dimensions to be used in the object detection model. Table 2 presents the default anchor boxes and generated anchor boxes for aortic valve detection, respectively.

With these three significant improvements, the number of parameters of the model, the detection time per image during the test, and the number of floating-point operations per second (GFLOPs) were found to be significantly reduced.

**Fig. 5:**
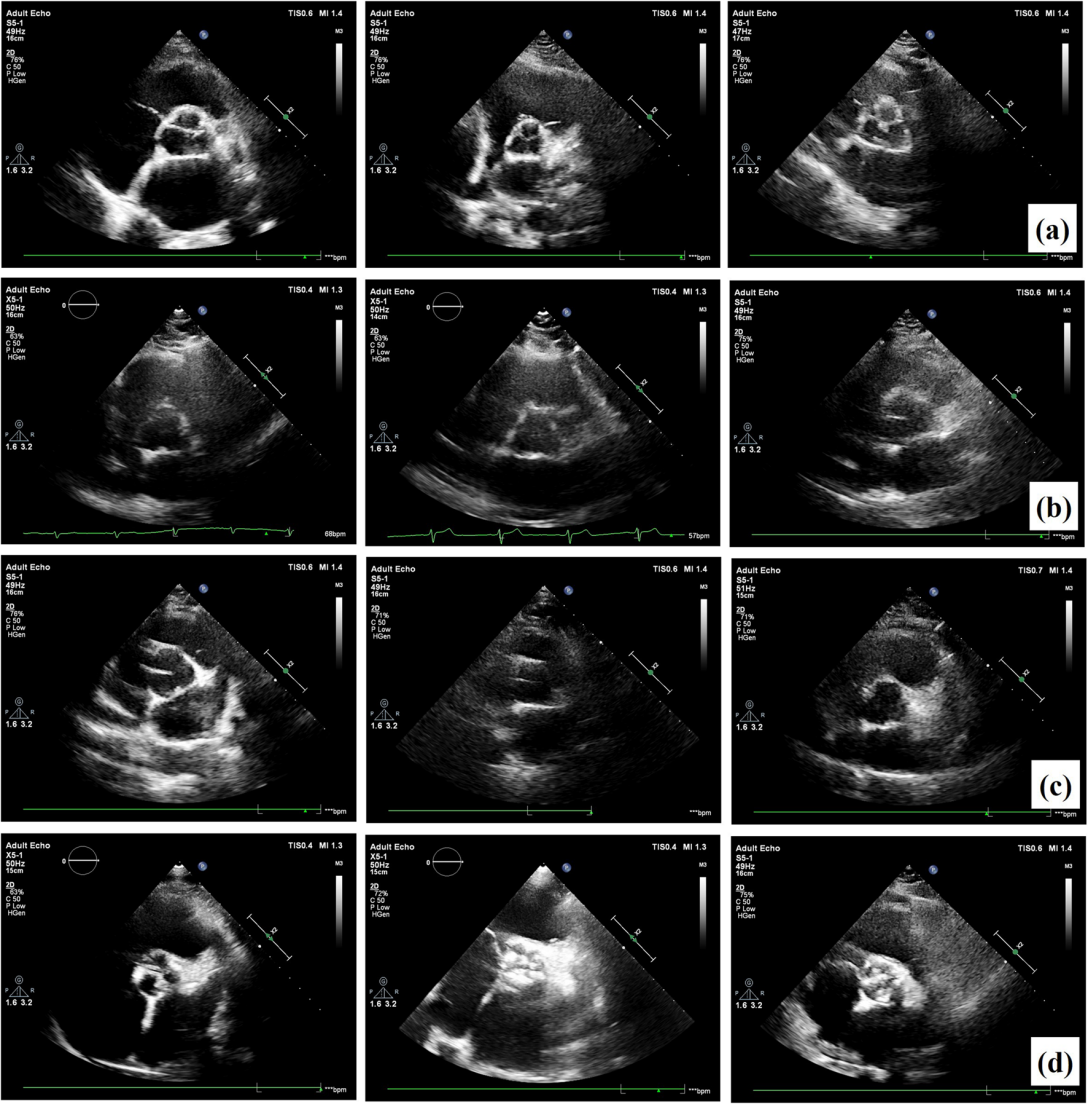
Some sample echocardiography images illustrating different conditions **a** diastole, **b** systole, **c** normal tissue, and **d** abnormal tissue

### Training details

In the network training, new anchor boxes generated to detect the aortic valve region were used instead of the default anchor boxes. Therefore, all models were trained from scratch. The models have trained with the stochastic gradient descent (SGD) optimization algorithm to the point where the validation loss did not change and tested on the same test dataset for fair comparison. All experiments were performed on Google Colaboratory (Colab) based on the Jupyter Notebook. Colab offers the possibility to use a Tesla K80 GPU and 12 GB RAM. All modifications were performed on the YOLOv5-s (small) model (yolov5s.pt), one of the sub-versions of YOLOv5. The parameters and their values were determined as epoch number of 500, batch size of 16, input image size of 768, momentum coefficient of 0.937, weight decay value of 0.0005, and learning rate of 0.01. The IOU threshold, which is the test parameter, was set as 0.9. When determining the input size, it was taken to ensure that the data set was sized appropriately to minimize information loss. The model training was started for each version with the specified parameters and the best results obtained at the end of the training and during the training were saved as a weight file. All other settings were left at default values (specified in the YOLOv5 file “hyp.scratch-low.yaml”).

**Fig. 6:**
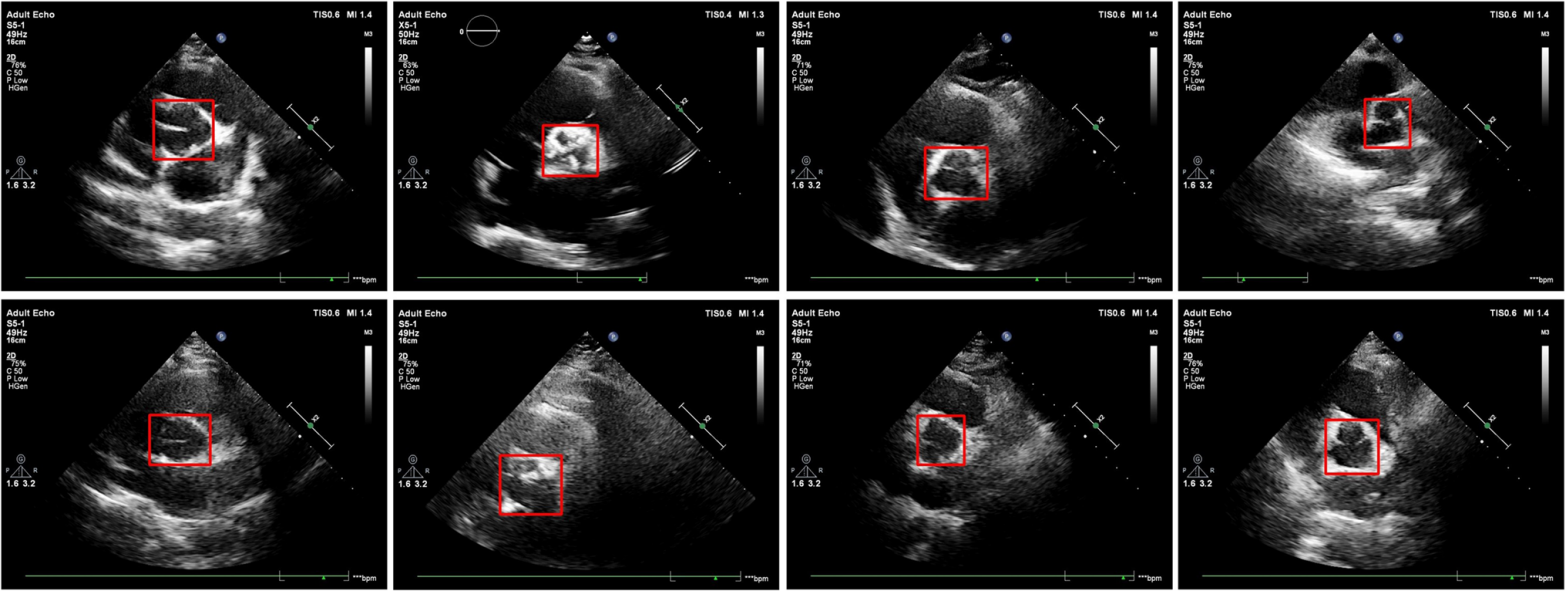
Sample images from the proposed aortic valve dataset. Red bounding boxes show the aortic valve regions of interest labeled by an expert cardiologist

## Results

### Aortic valve detection dataset

Several datasets have been proposed in the literature, each with different settings. However, there is no publicly shared dataset. Therefore, this study proposes a novel dataset for aortic valve detection. The dataset consists of 260 parasternal short-axis echocardiography images from 169 different patients at Karadeniz Technical University, Faculty of Medicine, Department of Cardiology. For the standardization of echocardiography images from all patients, the section of the parasternal short-axis image in which all three aortic leaflets were seen most clearly at the same time was identified and taken by the two expert cardiologists.

It should be noted that the appearance of the aortic valve varies significantly depending on whether it is in diastole, systole, normal, or abnormal (calcific) tissue conditions. This diversity complicates the automated detection of the aortic valve region of interest (ROI) from echocardiography images. However, it is important to emphasize that our dataset is robust as it includes all of the conditions mentioned. This demonstrates our ability to achieve accuracy in aortic valve detection under different conditions. In addition, we have provided example images corresponding to each condition in Fig. 5. As can be seen in Fig. 5d, the detection of the ROI becomes more difficult when calcification is present on the leaflet compared to the normal images shown in Fig. 5c. This is because in some images, the calcified area extends towards the aortic wall, making it difficult to determine the shape of the main structure. In addition, it may be easier to identify the ROI during diastole, as shown in Fig. 5a, than during systole, as shown in Fig. 5b, because the diastole phase often provides a clearer boundary for the region of interest.

Labeling was performed by the two expert cardiologists using Matlab 2022a Image Labeler image labeling toolbox. The Matlab image labeling tool provides a quick and easy way to annotate images by drawing regions of interest that can be assigned ROI labels. All images in the dataset were labeled by two expert cardiologists. Figure 6 shows the aortic valve regions of interest labeled by an expert cardiologist with rectangular label type in sample echocardiography images of the dataset.

The bounding box is a vector in [x, y, width, height] format, and the components of this vector are the horizontal and vertical coordinates of the upper left corner of the aortic valve region of interest, the width and height of the aortic valve region of interest, respectively. These labels are saved as.txt files in PASCAL VOC or YOLO format. Since the labels of the ImageNet and MS COCO datasets are saved in PASCAL VOC format, the labels obtained with the LabelImg tool in this study were saved in PASCAL VOC format. The bounding box in the image corresponds to the row of data stored in the corresponding.txt file. These numeric values in the row represent the information of the bounding box on the image. They represent (a) class name, (b) normalized center *x* coordinate, (c) normalized center *y* coordinate, (d) normalized width, and (e) normalized height, respectively. The values of the bottom left corner coordinate $(x_1,y_1)$, top right corner coordinates $(x_2,y_2)$, image width *w*, and height *h* of the bounding box were calculated as given in Eqs. (7–9).

7 $$\begin{aligned} b= & {} \frac{(x_{1}+x_{2})/2.0}{w} \end{aligned}$$

8 $$\begin{aligned} c= & {} \frac{(y_{1}+y_{2})/2.0}{h} \end{aligned}$$

9 $$\begin{aligned} d= & {} \frac{x_{2}-x_{1}}{w} \end{aligned}$$

10 $$\begin{aligned} e= & {} \frac{y_{2}-y_{1}}{h} \end{aligned}$$

The data set is divided into three parts training, validation, and test in the ratio of 80%-5%-15%. The training and validation set is used to perform deep network parameter tuning. Then, testing is performed to obtain the performance of the proposed AVD-YOLOv5 model.

**Table 3:** The effect of removing the small scale from YOLOv5-s on the performance

#scales	#layers	#parameters	Precision	Recall	mAP	GFLOPs	Inference
							time (ms)
3-scale	214	7,022,326	0.965	0.871	0.903	15.9	7.95
YOLOv5-s							
2-scale	191	5,240,036	0.96	0.903	0.961	14.5	6.15
YOLOv5-s							

### Evaluation metrics

In this study, we used four metrics, namely mean precision (mAP), accuracy, recall, and F1-score, to quantitatively evaluate the performance of the proposed model in detecting the aortic region of interest. Average precision (AP) is calculated for each class individually as the average of the precision values corresponding to different sensitivity values on the precision-sensitivity curve. Mean average precision (mAP) refers to the average of the AP values for all classes. Precision measures the ratio of correctly predicted positive samples to all predicted positive samples. A higher precision means that the model has fewer false positives. Recall, also known as sensitivity, measures the ratio of correctly predicted positive instances to all actual positive instances. A higher recall means that the model detects more true positive instances. F1-score, a harmonic mean of precision and recall, aims to find a balance between precision and recall. It is a single value that provides an overall evaluation of the model’s performance. These metrics are calculated as given below.

11 $$\begin{aligned} AP= & {} \int _{0}^{1}P(R)dR \end{aligned}$$

12 $$\begin{aligned} mAP= & {} \frac{\sum _{i=1}^{N}AP_{i}}{N} \end{aligned}$$

13 $$\begin{aligned} Precision= & {} \frac{TP}{TP+FP} \end{aligned}$$

14 $$\begin{aligned} Recall= & {} \frac{TP}{TP+FN} \end{aligned}$$

15 $$\begin{aligned} F1-score= & {} \frac{2 \times Precision \times Recall}{Precision + Recall} \end{aligned}$$

where TP, TN, FP, and FN represent the number of true positives, true negatives, false positives, and false negatives, respectively. *P*, *R*, and *N* values represent the precision, sensitivity, and total number of classes in all categories, respectively.

**Fig. 7:**
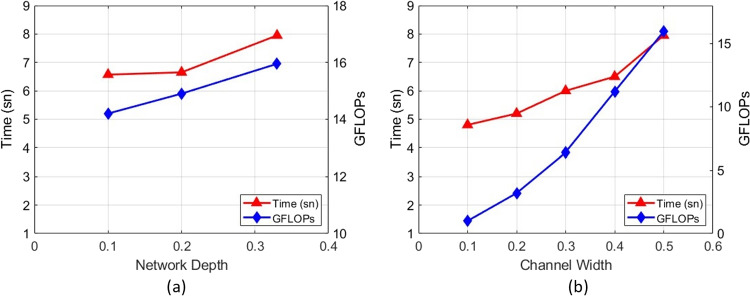
The effect of **a** depth and **b** channel width parameter on network structure

**Table 4:** The effect of network depth parameter in network architecture

Network	#layers	#parameters	Precision	Recall	mAP	GFLOPs	Inference
depth							time (ms)
0.1	193	6,652,406	0.818	0.87	0.926	14.2	6.57
0.2	200	6,816,758	0.931	0.903	0.909	14.9	6.65
0.3	214	7,022,326	0.952	0.871	0.903	15.95	7.95

In this study, to comprehensively assess the performance of the model, additional metrics were employed. These metrics provided insights into in terms of computational complexity and speed of the model. First, the GFLOPs (Giga Floating-Point Operations Per Second) metric was used to show how many billion floating-point operations the model can perform per second. GFLOPs also show how computationally intensive the model processes are and how fast these processes can be executed. In addition, the inference time metric was used to measure the time it takes for the model to process an image and generate detections. Low inference time values indicate that the model performs better in real-time applications requiring fast response.

### Ablation studies

To evaluate the proposed AVD-YOLOv5 model and validate its performance, several ablation studies were carried out with different parameter settings, including the following changes: (1) removing small scale from the configuration file, (2) changing the network depth and channel width, (3) decreasing the number of channels in convolution layers, (4) changing the number of the CSP BottleNeck layer, and (5) adding depth-wise separable convolution. Making these changes in various components, we also aimed to obtain an efficient and high-speed network architecture with higher detection accuracy.

**Removing small scale from the configuration file** In the head component of YOLOv5, there are three different sizes of scales used to transfer the predictions made in the last step to the output. These scales allow the network to better detect large, medium, and small details in the input images. Since the aortic region of interest in the ultrasound dataset does not contain extremely small details compared to the entire input image size, the small scale was removed from the network architecture. As can be seen in Table 3, the effect of removing the small scale on the results is that the number of parameters is reduced from nearly 7 to 5 million, the inference time per image is reduced from 7.95 to 6.15 ms, and the number of floating-point operations (GFLOPs) are reduced from 15.9 to 14.5.

**Changing the network depth and channel width** In YOLOv5, the depth parameter in the YAML configuration file is a scaling factor that controls the overall depth of the model and determines the number of layers in the YOLOv5 model. It is a percentage parameter that specifies how much of the total number of channels is involved in the calculations. It is applied to the number of modules specified in the model architecture. It scales the number of modules to achieve a shallower or deeper network. A value less than 1.0 (e.g., 0.33) reduces the number of layers for depth and results in a shallower network. A value greater than 1.0 for depth (e.g., 1.5) increases the number of layers, leading to a deeper network. To set the depth of the network, it is multiplied by the number of layers defined in the model architecture. For example, if the depth is set to 0.33, it means that the total number of layers in the model will be reduced to about one-third of the original number. Therefore, reducing the ratio of this parameter significantly reduces the complexity of the model, memory usage, inference time, the number of layers, and the number of parameters used for training and testing.

In this study, the “depth_multiple” parameter, which controls the depth of the network in the architecture, was tested with three different values for the small version. Depending on the depth parameter, the total number of floating-point operations and the inference time are shown in Fig. 7a. Table 4 also shows how changing the depth parameter affects the other parameters in the network and the performance. As seen in Fig. 7a and Table 4, when the value of “depth_multiple” is decreased, the inference time and the number of floating-point operations decreased. In this way, positive contributions to the improvement of the model performance were observed. It was decided to choose the depth value of 0.2 since there was not much difference in terms of the inference time of the model and the precision value was better. As a result of the experiments, the depth of the proposed AVD-YOLOv5 model was controlled by setting the depth parameter to an optimum value, and a balance between model performance and computational efficiency was established according to the specific use case and resource constraints.

The channel width is a percentage parameter that specifies how much of the total number of channels will be involved in the computations. This parameter, called width_multiple in the configuration file, controls the channel width of the layers in the model and is a scaling factor that determines the number of channels (also called width) in the layers of the YOLOv5 model. At runtime, the model is applied to the number of channels in each layer defined in the architecture. It scales the number of channels to set the overall width of the network, multiplied by the number of channels defined in each layer to set the width of the network. For example, a value less than 1.0 for width_multiple (e.g. 0.50) reduces the number of channels, resulting in a shallower network. In a network structure with a network width of 0.50, the channels through which the input image is processed are half the width of all channels in the architecture. This is useful for reducing model complexity, memory usage, or computational requirements. Conversely, a value greater than 1.0 (e.g., 1.50) for width_multiple increases the number of channels, leading to a deeper network. This can potentially improve the representational capacity of the model to capture more complex features, but can also increase computational demands.

In this study, the “width_multiple” parameter, which controls the channel width in the network architecture, was tested with five different values for the small version, and the change in the total number of multiplication operations and test time depending on the width parameter is given in the Fig. 7b. Table 5 also shows how changing the width parameter affects the other parameters in the network and the performance. As seen in Fig. 7b and Table 5, when the ratio of this parameter “width_multiple” is decreased, the number of layers and parameters used for training and testing is significantly reduced. As a result, the inference time is reduced from 7.95 to 4.8 ms, and the number of floating-point operations is reduced from 15.95 to 1. Therefore, considering the acceptable performance and model size, the network channel width is set to 0.1.

**Table 5:** The effect of channel width parameter in network architecture

Channel	#layers	#parameters	Precision	Recall	mAP	GFLOPs	Inference
width							time (ms)
0.1	214	328,622	0.926	0.871	0.909	1	4.8
0.2	214	1,190,214	0.895	0.871	0.893	3.2	5.2
0.3	214	2,685,326	0.932	0.903	0.913	6.4	6.0
0.4	214	4,652,070	0.95	0.871	0.92	11.2	6.5
0.5	214	7,022,326	0.952	0.871	0.903	15.95	7.95

**Table 6:** The effect of the number of channels in convolution layers in network architecture

#channels in	#layers	#parameters	Precision	Recall	mAP	GFLOPs	Inference
convolution layers							time (ms)
8768	214	7,022,326	0.965	0.871	0.903	15.9	7.95
4672	214	1,853,814	0.89	0.9	0.926	4.9	5.40

**Fig. 8:**
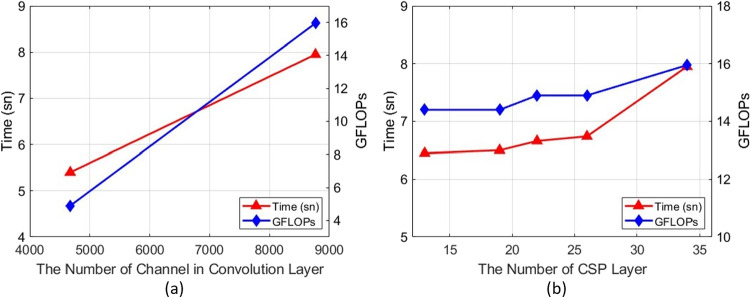
The effect of the number of channels in the convolution layer (**a**) and the number of CSP BottleNeck blocks (**b**) on the network structure

**Decreasing the number of channels in convolution layers** The convolution layer is used to extract feature maps from input data, which is one of the most fundamental stages of deep learning. Reducing the number of channels in the network architecture of convolution layers is advantageous in terms of parameters and testing time, but disadvantageous for model memory. Fewer convolution layer channel sizes require fewer parameters and less computational power for the model, thus reducing the memory and computational power of the model. This allows the model to run faster, but it can also limit its ability to learn more complex features which means that the model will have less learning capacity. It also limits the model’s ability to learn simpler and lower-complexity data patterns. For more complex data sets, this can cause the model to be inefficient. On the other hand, reducing the number of channels in the convolution layer speeds up the training and testing phases. This is useful for faster processing of large data sets or to meet the real-time requirements of the application. To observe all these effects on the network, the model was retrained by halving the channel size of all convolution layers except for the focal convolution, which is at the top of the spine and head components. As shown in Table 6 and Fig. 8a, halving the convolution channel size reduces the number of parameters used in training, the number of multiplication operations, and the test time. However, this process also reduces the memory and learning ability of the model, so it is observed that the precision value is reduced proportionally too much. For these reasons, considering the performance and efficiency of the model, it was decided not to halve the channel size of the convolution layers in the main architecture.

**Changing the number of the CSP BottleNeck block** The CSP BottleNeck module used in the spine and neck components, basically called C3, is detailed in Fig. 2. It is a structure that includes CBL, BottleNeck, Convolution (Conv), and Concatenation (Concat) blocks. CBL consists of Convolution (Conv), Batch-Normalization (BN), and Activation Function (Sigmoid Leaky-Relu, SILU) blocks. BottleNeck, on the other hand, consists of two CBL blocks and a residual skip connection. Therefore, any change to the CSP_BottleNeck block indirectly affects many sequential connections. The CSP_BottleNeck structure works based on divide-and-conquer logic. It divides the whole input into parts of certain proportions, processes each of them internally, and then combines them according to a certain hierarchy. This reduces the computation time, reduces the size of the images, and makes them more dense in terms of expressive meaning. To observe the effect of the CSP blocks in the network on the value parameters, experiments were performed by reducing the number of all CSP blocks (CSP1_X and CSP2_X) in the architecture. As can be seen in Fig. 8b, reducing the number of CSP blocks reduced the number of floating-point operations and inference time as it reduced the proportion of multiple nested structures. According to the results presented in Table 7, reducing the number of CSP blocks accelerated the training and testing time while maintaining the success in the accuracy (precision) value. Considering the changes made and their effects on the network, it was decided to reduce the number of CSP BottleNeck blocks in the backbone and head components by 1/3 to 1,2,3,3,3,3,3,3 respectively, since it did not cause a significant decrease in the precision value while improving the performance of the model.

**Table 7:** The effect of the number of CSP BottleNeck layers in network architecture

#CSP Layers	#layers	#parameters	Precision	Recall	mAP	GFLOPs	Inference time (ms)
3,6,9,3 3,3,3,3	214	7,022,325	0.952	0.871	0.903	15.95	7.95
2,4,6,2 3,3,3,3	200	6,816,758	0.818	0.87	0.909	14.7	6.76
2,4,6,2 2,2,2,2	200	6,816,756	0.818	0.87	0.909	14.7	6.74
1,2,3,1 3,3,3,3	193	6,652,406	0.931	0.903	0.926	14.2	6.66
1,2,3,1 1,1,1,1	193	6,652,406	0.931	0.903	0.926	14.2	6.58

**Adding depth-wise separable convolution** Depth-wise separable convolution (DS-Conv) differs from standard convolution (Conv) in that it first divides the input image into groups of the desired output size and then performs filter traversal for each of them. This grouping process reduces the size of the image. Then, a kernel size is selected according to the output size obtained in the point-wise part and standard convolution is applied. As can be seen in Table 8, the inference time per image is significantly reduced from 7.95 to 5.8 and the number of floating-point operations is reduced from 15.9 to 11.2. The precision value has no significant disadvantage (0.012) in terms of precision compared to the standard convolution. It also significantly improved the recall value from 0.871 to 0.997.

### Results and discussion

#### Detection results

In this study, the limitations of existing YOLO-based detection methods, in particular their high computational complexity and resource requirements, are discussed in detail. Since these methods are usually trained on very large datasets, they require large and complex models, which leads to practical difficulties in real-world applications. Especially in sensitive application areas such as medical imaging, the usability and effectiveness of such complex models are often limited. On the other hand, it is very important to emphasize the requirements of lightweight models. Lightweight models have the potential to achieve similar or better performance using less computational power and memory. This makes them more practical for use in resource-constrained environments such as mobile devices or cloud-based applications. Furthermore, the faster training and deployment of lightweight models provide a significant advantage for rapid prototyping and innovative research. In conclusion, the lightweight model proposed in this work represents an important step towards overcoming the limitations of current detection methods and providing more effective solutions in medical imaging areas such as aortic valve detection. The advantages offered by lightweight models have the potential to replace the complex models widely used in such applications.

**Fig. 9:**
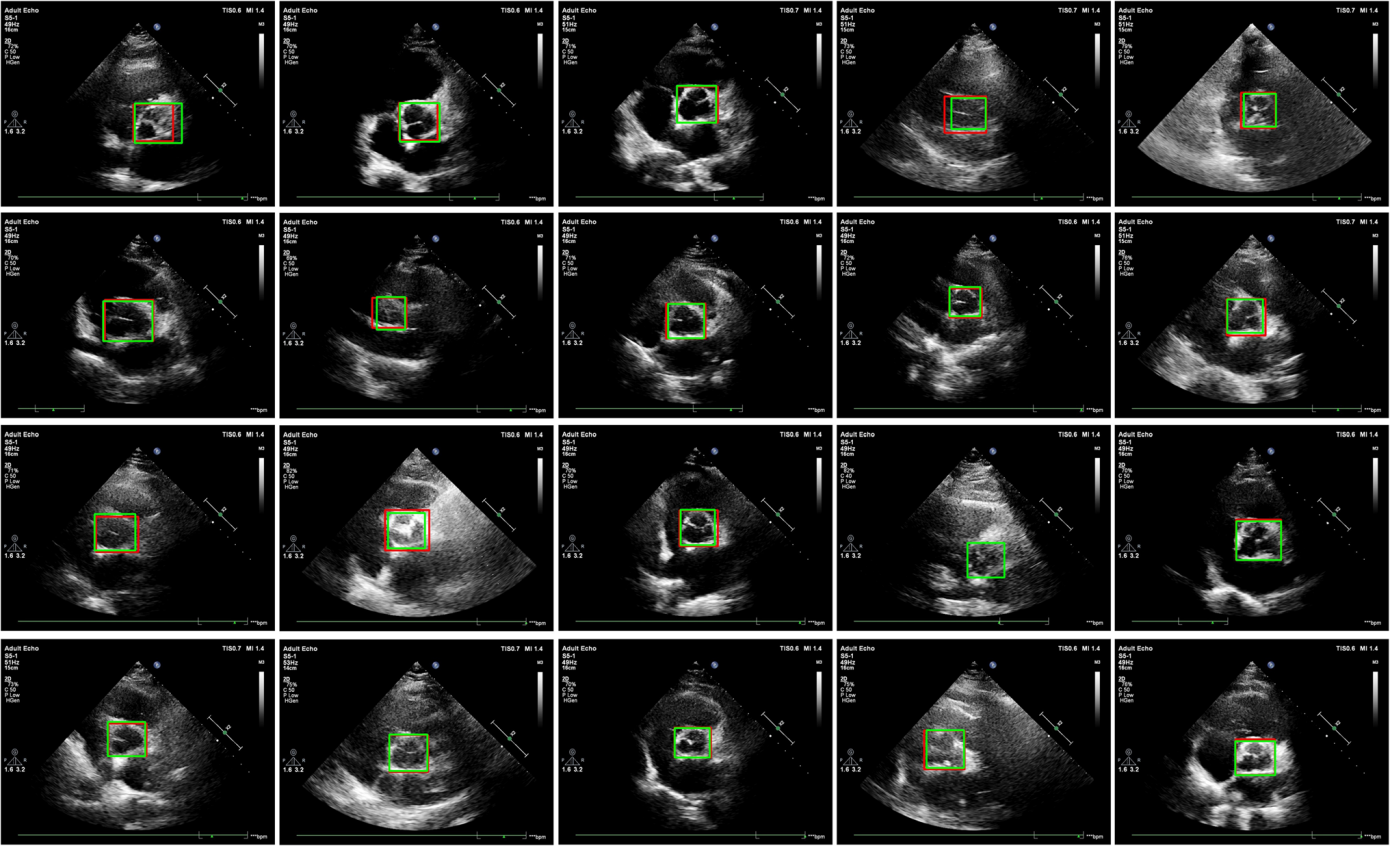
Aortic valve detection results from different echocardiography images. The green and red bounding boxes denote the ground truth and detected aortic valve regions, respectively

The performance of the AVD-YOLOv5 model is evaluated both qualitatively and quantitatively. Table 9 summarizes the quantitative performance comparison results of the proposed method with four state-of-the-art YOLOv5 model sub-versions, namely YOLOv5-s (small), YOLOv5-m (medium), YOLOv5-l (large), and YOLOv5-x (xlarge). Figure 9 qualitatively shows the aortic valve detection results of some sample images in the proposed aortic valve dataset. As shown in Fig. 9, aortic valves with different shapes and scales can be detected with very high precision. This shows that the proposed AVD-YOLOv5 detection model has very strong robustness.

**Table 8:** The effect of the convolution method used in network architecture

Convolution	#layers	#parameters	Precision	Recall	mAP	GFLOPs	Inference time (ms)
Method							
Conv	214	7,022,325	0.952	0.871	0.903	15.9	7.95
DS-Conv	214	4,697,910	0.94	0.997	0.988	11.2	5.8

**Table 9:** Performance comparison of the proposed AVD-YOLOv5 model with four state-of-the-art YOLOv5 model sub-versions

Method	#layers	#parameters	GFLOPs	Precision	Recall	mAP	Inference time (ms)
YOLOv5-x	445	86,217,814	203.8	0.8	0.898	0.868	16.76
YOLOv5-l	368	46,138,294	107.6	0.957	0.903	0.926	9.72
YOLOv5-m	291	20,871,318	47.9	0.809	0.871	0.87	8.42
YOLOv5-s	214	7,022,326	15.8	0.952	0.871	0.903	7.95
AVD-YOLOv5	170	148,844	0.6	0.943	0.868	0.896	2.58

**Fig. 10:**
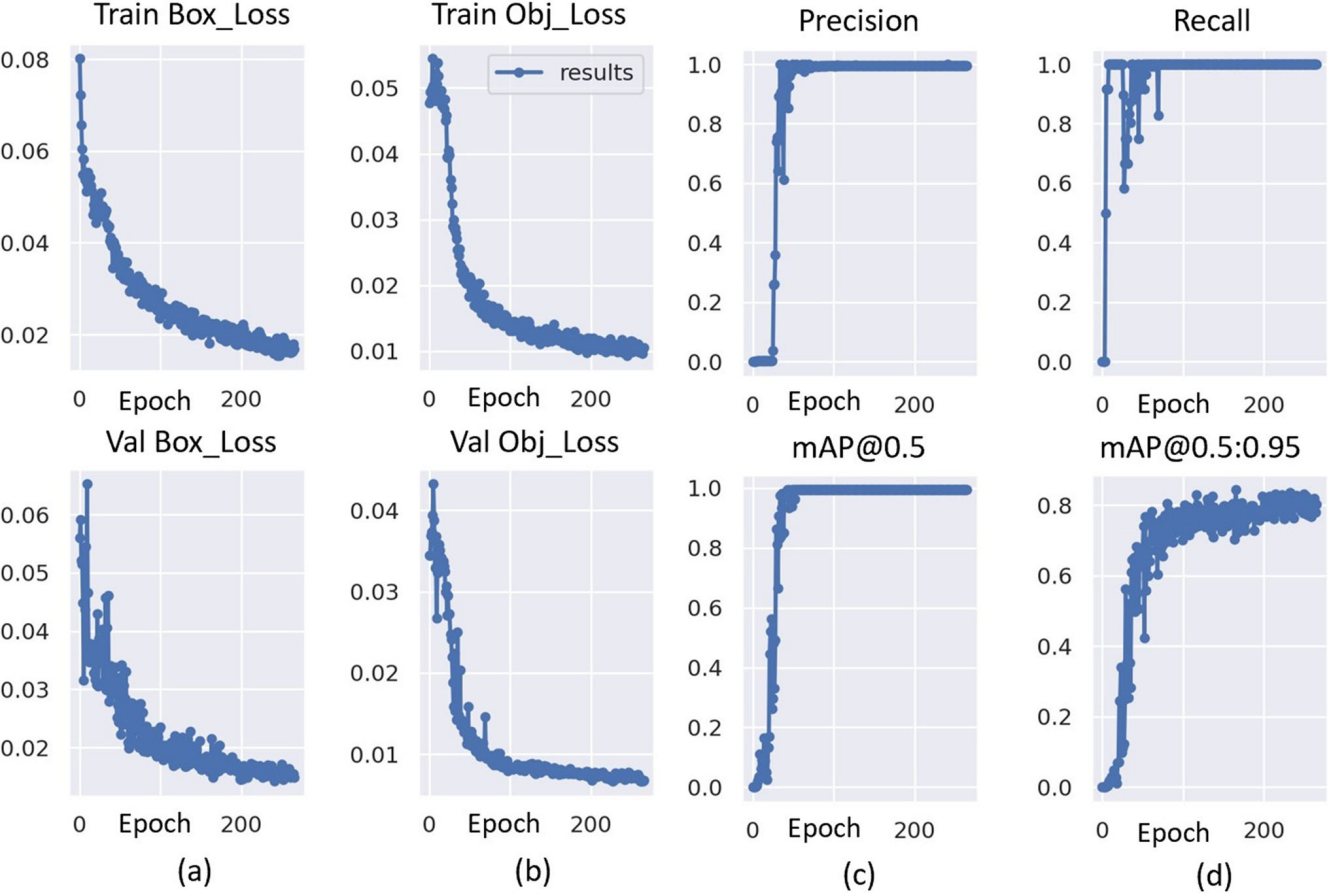
The training graphs of the proposed AVD-YOLOv5 model. **a** Box loss, **b** objectness loss, **c** precision, and **d** recall

From Fig. 9 and Table 9, we can draw the following conclusions:

- The proposed AVD-YOLOv5 model has significantly fewer layers (170), parameters (148,844), and GFlops (0.6) compared to other YOLOv5 sub-versions. This suggests a more lightweight architecture in the medical field where high-speed diagnosis is crucial. AVD-YOLOv5 has the least number of network parameters (148,844), while the number of network parameters of YOLOv5-s (7,022,326) is five times more than our method. This leads to higher training efficiency and faster detection speed. AVD-YOLOv5 has the lowest computational cost with 0.6 GFLOPs, about 15 times lower than YOLOv5-s (15.8).
- Choosing a low IOU threshold value increases the probabilistic probability that the recognized object is the searched object, thus increasing the precision value, but as the IOU threshold value decreases, the reliability and robustness of the model decrease. In general, when testing object recognition architectures in medical and other fields, the IOU threshold value, which is one of the values indicating reliability, is taken between 0.5 and 0.7 [25] [26] [27], while 0.9 is taken for the AVD-YOLOv5 model. All comparisons are based on the results obtained at the IOU threshold value of 0.9. Despite the high threshold value, it was observed that very successful results were obtained.
- In terms of precision, the AVD-YOLOv5 model performs well with a precision of 0.943. This indicates a low false positive rate. The recall value is also high as with the other models, indicating a good performance in capturing true positive samples. These results demonstrate the ability of the proposed model to perform efficient and fast detection without performance loss.
- The mAP values are relatively close among the models, with the AVD-YOLOv5 model achieving a mAP of 0.896. This demonstrates consistent accuracy in object detection.
- The inference time for the AVD-YOLOv5 model (2.58 ms) is significantly lower compared to other YOLOv5 sub-versions. The results show that the proposed model is about six times faster than the YOLOv5-x model (16.76 ms) and about three times faster than the YOLOv5-s model (7.95 ms).

**Table 10:** Performance comparison of the proposed AVD-YOLOv5 model with similar studies in the literature

Reference	IoU	Method	Accuracy	Precision	Recall
	threshold				
Nizar et al. [12]	0.5	Faster R-CNN + Inceptionv2	0.949	0.942	0.957
		ResNet50 + Faster R-CNN	0.724	0.668	0.89
Hatfaludi et al. [14]	0.5	VGG-16	−	0.894	0.988
		VGG-19	0.852	0.997	
		ResNet-19	0.894	0.997	
		ResNet-50	0.932	**0**.**997**	
Proposed method	**0**.**9**	AVD-YOLOv5	**0**.**95**	**0**.**943**	0.868

The values in bold indicate the best results

#### Graphical results of training

The training graphs of the AVD-YOLOv5 architecture, which we reconfigured for object detection, are also shown in Fig. 10. The graphs show that as the number of epochs increases, the precision and recall values gradually increase and the loss values of the validation and train stages gradually decrease. In addition, the convergence time of precision and recall values to 1 is quite short and the convergence time of loss values to 0 is proportional to the number of epochs. Considering all these data, it can be said that the model was successfully trained in a short time.

#### Comparison to established similar CNN-based models

To further assess the suitability of the proposed model, we compared our proposed model (AVD-YOLOv5) with state-of-the-art CNN-based studies conducted for aortic region detection in echocardiography images, as summarized in Table 10. These studies were carried out on different datasets with very small IoU threshold values. Additionally, due to the unavailability of shared datasets, a fair and rigorous comparison could not be achieved by using identical configurations and settings for all models. In the study by Nizar et al. [12], the Faster R-CNN and Inception V2 models achieved an accuracy of 94.9%, precision of 94.2%, and recall of 95.7%. However, it is noteworthy that a relatively low IoU threshold value (0.5) was used in this study. Similarly, in the study by Hatfaludi et al. [14], the ResNet-50 model yielded the highest precision of 93.2% and recall of 99.7%, with a low IoU threshold value of 0.5. Our proposed study, on the other hand, demonstrated comparable results with an IoU threshold value as high as 0.9, achieving accuracy of 95%, precision of 94.3%, and recall of 86.8%. The IoU threshold value ensures accurate detection of searched objects. Therefore, the results presented in our proposed work are more reliable considering the high IoU threshold value. It is important to note that choosing a low IoU threshold increases the probability that the recognized object is the searched object and thus improves sensitivity by increasing the number of TP and improves recall by decreasing the number of FN. The low recall value in our study is due to this fact.

Overall, the proposed AVD-YOLOv5 model seems to strike a balance between accuracy and speed and offers a competitive performance with significantly fewer parameters and computational complexity. It seems to be a promising solution for object detection tasks where both high accuracy and efficiency are expected.

## Conclusion

In this study, a new lightweight network architecture, AVD-YOLOv5, is proposed for the high-speed aortic valve detection problem. It provides several improvements to the original YOLOv5 architecture. Small-scale detection was removed from the configuration file, as the aortic region of interest does not contain excessively small details relative to the size of the whole input image. Depth-wise separable convolution was used in the CSP and consequently CBL blocks, different from the standard convolution. The problem-specific anchor boxes that best fit the searched object (aortic valve) were calculated and used. Experiments and comparisons are carried out on a new and larger dataset created for aortic valve detection in echocardiography images. The results show that the proposed method can perform much more efficient and faster detection with similar accuracies as other state-of-the-art existing methods with minimum parameters. This approach holds the potential for transfer to other medical detection domains in future research. Moreover, the dataset will undergo further refinement by annotating additional normal (non-calcified) and abnormal (calcified) images. Additionally, future efforts will focus on classifying the aortic valve regions of interest into non-calcified or calcified categories. This advancement aims to strengthen the effectiveness of our proposed method and potentially facilitate more precise and efficient diagnosis and treatment planning for conditions like aortic stenosis, based on valve anomalies.

## Data Availability

The dataset created in this paper is being further developed, so the dataset is not currently available.
